# Expression and clinical significance of PD-L1 and infiltrated immune cells in the gastric adenocarcinoma microenvironment

**DOI:** 10.1097/MD.0000000000036323

**Published:** 2023-12-01

**Authors:** Qiuying Quan, Lingchuan Guo, Lili Huang, Zhiju Liu, Tianwei Guo, Yu Shen, Sisi Ding, Cuiping Liu, Lei Cao

**Affiliations:** a Department of Pathology, The First Affiliated Hospital of Soochow University, Suzhou, Jiangsu, China; b Department of Clinical Laboratory, Children’s Hospital of Soochow University, Suzhou, Jiangsu, China; c Department of Pathology, Changshu Hospital of Affiliated to Nanjing University of Chinese Medicine, Changshu, Jiangsu, China; d Jiangsu Institute of Clinical Immunology, The First Affiliated Hospital of Soochow University, Suzhou, Jiangsu, China; e Jiangsu Key Laboratory of Clinical Immunology, Soochow University, Suzhou, Jiangsu, China; f Jiangsu Key Laboratory of Gastrointestinal Tumor Immunology, Suzhou, Jiangsu, China.

**Keywords:** gastric adenocarcinoma, prognosis, programmed death-ligand 1 (PD-L1), tumor immunity, tumor-infiltrating immune cells

## Abstract

Programmed death-ligand 1 (PD-L1) is a crucial negative costimulatory molecule expressed on both tumor and immune cells. It binds to programmed death-1, facilitating tumor escape. Tumor-infiltrating immune cells play a vital role in this process. However, the clinical relationship between PD-L1 expression and tumor-infiltrating immune cells remains uncertain. Immunohistochemistry (IHC) was utilized to assess PD-L1 expression and TIIC markers (CD3, CD4, CD8, CD19, CD31, CD68, CD11c, CD56, and α-smooth muscle actin) in gastric adenocarcinoma tissues from 268 patients. The aim was to explore the prognostic significance of PD-L1 and the infiltration of different immune cell types. The study analyzed overall survival and the correlations between PD-L1 expression, immune cell infiltration, and clinicopathological characteristics. Among the 268 patients, 52 (19.40%) exhibited high PD-L1 expression on tumor cells (TPD-L1), while 167 (62.31%) displayed high PD-L1 expression on immune cells (IPD-L1). Patients with high IPD-L1 expression showed improved survival compared to those with low IPD-L1 expression (*P* = .028). High TPD-L1 expression associated with various clinicopathological features, such as larger tumor size, poorer differentiation, deeper invasion depth, and higher tumor stage. Conversely, patients with high IPD-L1 expression exhibited shallower tumor invasion and lower mortality rates. Univariate analysis indicated that superficial tumor infiltration, absence of lymph node and distant metastasis, low tumor stage, high IPD-L1 expression, and elevated CD8 and CD19 expression were associated with a reduced risk of tumor progression. Multivariate analysis revealed that patients with high IPD-L1 and CD8 expression or high TPD-L1 and low CD31 expression experienced significantly better overall survival than patients with other combinations. The findings indicate that patients with high PD-L1 expression in immune cells have a substantially improved prognosis. Additionally, the combination of PD-L1 with CD8 or CD31 expression status can serve as an indicator of prognosis in patients with gastric adenocarcinoma.

## 1. Introduction

Gastric carcinoma (GC) ranks among the top 5 most malignant cancers globally and is the second most lethal cancer, with adenocarcinomas constituting over 95% of GC cases. In China, GC stands as the third most common malignant tumor.^[1–4]^ Traditional treatments like surgery, radiation therapy, and chemotherapy have shown some improvement in patient survival, but overall survival (OS) remains largely unaffected. As a result, there is a growing interest in therapies targeting immunological checkpoints in GC patients. Monoclonal antibodies against programmed death-1 (PD-1) and programmed death-ligand 1 (PD-L1) have gained popularity due to their exceptional efficacy and minimal side effects.^[5,6]^

The interaction between PD-L1 and its receptor PD-1 induces negative regulatory functions, inhibiting the activation, proliferation, and cytokine secretion of T cells, which in turn facilitates immune escape of tumor cells.^[7,8]^ Significant progress has been made in using PD-1/PD-L1-specific monoclonal antibodies like nivolumab and atezolizumab for the treatment of various cancers, including GC, non-small cell lung cancer, malignant melanoma, and urologic epithelial tumors.^[6,8–12]^ However, the long-term efficacy of PD-1/PD-L1 inhibitors is observed in only 10% to 30% of patients, necessitating accurate patient selection to enhance therapeutic efficacy. Studies on tumors have suggested that the effectiveness of anti-PD-L1 treatment is related to the expression of PD-L1 in tumor tissues and infiltrating cells within the tumor stroma.^[13–15]^ Nevertheless, the therapeutic impact of PD-1/PD-L1 in various cancers remains a subject of debate.^[16–19]^ Thus, it is important to investigate the patterns of PD-L1 expression and tumor-infiltrating immune cells (TIICs) and their correlation with patient prognosis.

T lymphocytes, including CD8+ and CD4+ T cells, play a role in inducing tumor cell apoptosis through the secretion of cytotoxins and cytokines. B lymphocytes exhibit antitumor effects by differentiating into memory B cells or plasma cells that secrete antibodies. Innate immune cells, such as macrophages, dendritic cells, and natural killer cells, contribute to immune surveillance and cytotoxicity in the tumor immune microenvironment. Moreover, an increase in vascular endothelial cells and tumor-associated fibroblasts is considered a poor prognostic factor for patient survival due to immune escape regulation.^[20–23]^ As a result, different immune cells exert complex functions in the tumor microenvironment. While an association between PD-L1 expression and TIICs has been established, further research is needed to understand the relationship between PD-L1 expression and different types of TIICs in the context of specific clinicopathological characteristics.

In this study, we utilized a gastric adenocarcinoma tissue microarray (TMA) to investigate the correlation between PD-L1 expression and TIICs markers, as well as the impact of PD-L1 expression in combination with various immune cell indicators on OS. Our findings suggest that PD-L1 expression patterns and co-expression with different TIICs markers in GC may have implications for patient prognosis and serve as a valuable guide for potential clinical interventions.

## 2. Materials and methods

### 2.1. Patients and specimens

Samples from 268 patients with gastric adenocarcinoma (GAC) (210 males, 58 females), collected between 2011 and 2013, were retrieved from the Department of Pathology, The First Affiliated Hospital, Soochow University. In each case, the diagnosis of GAC was made by 2 experienced pathologists. Samples were obtained by surgical resection, preserved in neutral buffered formalin, embedded in paraffin, and stained with hematoxylin and eosin (H&E). Clinicopathological data were retrieved from all the patients through clinical and pathological systems. Follow-ups were done by telephone or outpatient review. The clinical features of 268 patients with GAC are summarized in Table S1, Supplemental Digital Content, http://links.lww.com/MD/K860. In this study, OS was defined as the time from treatment onset to death. Notably, OS data were available for only 198 patients.

### 2.2. Inclusion criteria and exclusion criteria

#### 2.2.1. Inclusion criteria.

(1) Patients after radical gastric cancer surgery;
(2) Histological diagnosis is gastric adenocarcinoma;
(3) Patients aged from 18 to 88-years-old;

#### 2.2.2. Exclusion criteria.

(1) Radiotherapy, chemotherapy, or immunotherapy were given before surgery;
(2) Patients who have a history of other tumors except for gastric adenocarcinoma;
(3) Patients have rheumatic immune system disease or lymphatic hematopoietic system disease;

### 2.3. TMA construction

TMA was constructed as previously described.^[24]^ Briefly, based on the H&E-stained slides, a skilled pathologist identified representative sites in 268 paraffin-embedded specimens. TMAs were created using automated tissue array equipment (Beecher ATA-27; Estigen OU, Tartu, Estonia). Tissue cores were positioned in the TMA blocks in accordance with the preplanned modules. The completed TMA blocks were sliced into 4-µm sections by an experienced pathology technician.

### 2.4. Immunohistochemistry

IHC staining was carried out in accordance with a previously established methodology.^[24]^ Paraffin sections were transferred to sticky slides and allowed to dry at 72 °C for 1 hour. After the sections were deparaffinized with xylene and rehydrated in successively increasing ethanol dilutions, endogenous peroxidase activity was blocked by the application of 0.3% hydrogen peroxide for 30 minutes. Antigen retrieval was performed by boiling the slides at 110 °C in a citrate buffer (pH 6.0) for 3 minutes. After blocking with 5% bovine serum albumin, the sections were incubated with an rabbit antihuman PD-L1 monoclonal (1:200; clone number E1L3N; CST), mouse antihuman CD3 monoclonal (ready to use; clone number LN10; ZSGB-BIO; China), mouse antihuman CD4 monoclonal (ready to use; clone number UMAB64; ZSGB-BIO; China), rabbit antihuman CD8 monoclonal (ready to use; clone number SP16; ZSGB-BIO; China), mouse antihuman CD11c monoclonal (1:50; clone number 5D11; ZSGB-BIO; China), mouse antihuman CD19 monoclonal (ready to use; clone number UMAB103; ZSGB-BIO; China), mouse antihuman CD31 monoclonal (ready to use; clone number UMAB30; ZSGB-BIO; China), mouse antihuman CD56 monoclonal (ready to use; clone number UMAB83; ZSGB-BIO; China), mouse antihuman CD68 monoclonal (ready to use; clone number KP1 ZSGB-BIO; China), mouse antihuman α-SMA monoclonal (ready to use; clone number UMAB237; ZSGB-BIO; China) at 4 °C overnight. The slides were then washed and incubated with horseradish peroxidase-labeled secondary antibodies (GP016129 anti-mouse and rabbit; Genetech, China) at room temperature for 30 minutes. After incubation, the samples were washed and the Dako EnVision detection system (Dako, Carpinteria, CA) was used for immunodetection. Slides were counterstained with Mayer hematoxylin and mounted with neutral glue. In the negative control, mouse or rabbit IgG were used in place of the primary antibody. The positive control was human tonsil tissue.

### 2.5. Assessment of tissue staining

All IHC slides were scanned as previously described.^[24]^ PD-L1 expression in tumor and immune cells was quantified using the tumor proportion score and immune proportion score, respectively. The tumor proportion score was calculated by dividing the number of PD-L1-positive tumor cells by the total number of live tumor cells and multiplying by 100%. The immune proportion score was defined as any intensity of PD-L1 membrane and cytoplasmic staining of tumor-associated immune cells divided by the total number of tumor-associated immune cells, and multiplied by 100%. High PD-L1 expression was defined as ≥50% for tumor cells and ≥10% for immune cells; low PD-L1 expression was defined as 0% to 49% for tumor cells and 0% to 9% for immune cells.^[25]^ All samples were confirmed to include at least 100 viable tumor cells, which were considered adequate for the assessment of PD-L1 expression. For the evaluation of tumor-infiltrating lymphocytes, the slides were stained with histoscore (0–3) as follows: 0, no staining; 1, <10% staining; 2, 10% to 50% staining; and 3, >50% staining. Sections with histoscores of 0 and 1 were defined as the low-expression group, and sections with histoscores of 2 and 3 were defined as the high-expression group. For instance, TPD-L1^high^CD8^high^ combination denotes patients with both tumor PD-L1-high and CD8-high expression. TPD-L1^high^CD8^low^ combination denotes patients who have both tumor PD-L1-high and CD8-low expression.

### 2.6. Statistical analysis

Statistical analyses were performed using SPSS 19.0 (IBM Corp., Armonk, NY). PD-L1 expression and clinicopathological characteristics were compared between the groups using chi-squared tests. Correlations between PD-L1 and TIICs marker expression were analyzed using Pearson correlation. When dealing with categorical data, the chi-squared test or Fisher exact test was used to examine the difference between the 2 groups. Kaplan–Meier analysis was used to estimate survival, and the curves were compared using log-rank tests. To calculate hazard ratios, both univariate and multivariate analyses were performed. In all analyses, *P* < .05 were considered statistically significant.

## 3. Results

**Figure 1.**
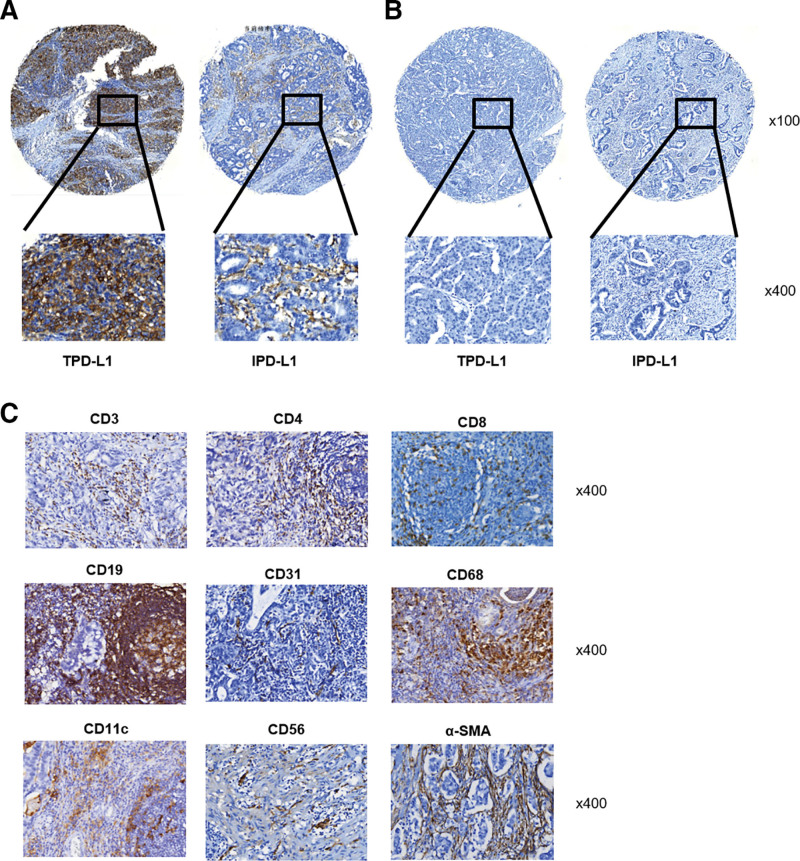
Immunohistochemical staining of PD-L1 and tumor infiltrated immune cells. (A) Representative pictures of PD-L1 high expression in tumor cells and in immune cells (upper ×100, lower ×400); (B) Representative pictures of PD-L1 low expression in tumor cells and in immune cells (upper ×100, lower ×400); (C) Representative pictures of CD3, CD4, CD8, CD19, CD31, CD68, CD11c, CD56, and α-SMA immunostaining (×400).

**Figure 2.**
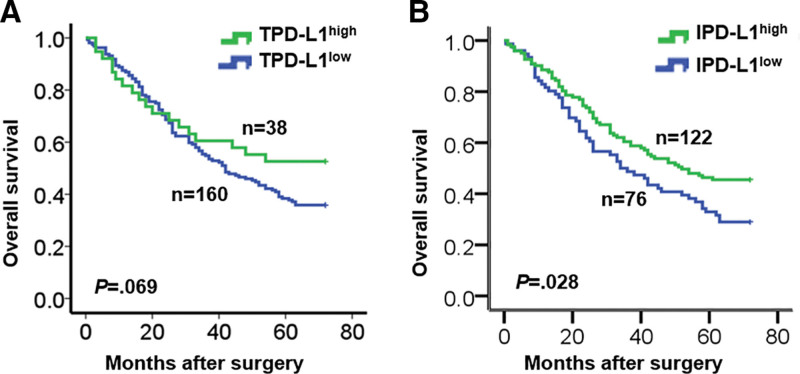
Overall survival difference of gastric cancer patients between high and low levels of (A) TPD-L1 expression or (B) IPD-L1 expression.

In 268 samples of GAC, PD-L1 was found to be expressed both in tumor cells and TIICs (Fig. 1A and B). In contrast, CD3, CD4, CD8, CD19, CD31, CD68, CD11c, CD56, and α-SMA showed scattered or focal expression primarily in immune cell membranes (Fig. 1C). The study aimed to determine the clinicopathological significance of PD-L1 expression in GAC tumors and TIICs by evaluating its relationship with clinicopathological parameters. PD-L1 expression was substantially increased in tumor cells in 52 (19.4%) patients and in TIICs in 167 (62.3%) patients (Table 1). PD-L1 expression on tumor cells (TPD-L1) had a negative prognostic impact and was significantly associated with tumor volume (*P* = .041), tumor differentiation *(P* = .001), tumor depth (*P* = .024), and tumor stage (*P* = .025; Table 1), but not with survival (*P* = .069; Fig. 2A). Conversely, IPD-L1 had a positive prognostic effect and was significantly associated with tumor depth (*P* = .001), patient mortality (*P* = .017; Table 1), and better survival (*P* = .028; Fig. 2B). Notably, TPD-L1 positively associated with tumor depth, while IPD-L1 negatively associated with tumor depth.

**Table 1:** Relationship between PD-L1 expression and clinicopathological features.

Clinicopathologic features	Total no	TPD-L1 expression		*P*	IPD-L1 expression		*P*
		Low	High		Low	High	
All cases	268	216	52		101	167	
Age				.315			.960
<70	164	129	35		62	102	
≥70	104	87	17		39	65	
Sex				.925			.060
Female	58	47	11		28	30	
Male	210	169	41		73	137	
Tumor volume (cm^3^)				.041			.765
<5	186	156	30		69	117	
≥5	82	60	22		32	50	
Tumor differentiation				.001			.236
Well	6	6	0		2	4	
Moderate	121	107	14		41	80	
Poor	141	103	38		58	83	
Tumor depth				.024			.001
T1	36	34	2		5	31	
T2 + T3 + T4	232	182	50		96	136	
LN involvement				.248			.057
N0	85	72	13		25	60	
N1 + N2 + N3	183	144	39		76	107	
Metastasis				.930			.500
M0	238	192	46		88	150	
M1	30	24	6		13	17	
Tumor stage				.025			.074
0 + I	43	40	3		11	32	
II + III + IV	225	176	49		90	135	
Death				.064			.017
No	78	58	20		22	56	
Yes	120	102	18		54	66	

The study also analyzed the relationships between TIICs expression and clinicopathological characteristics, and a significant association was found between the expression of several TIICs markers and the pathological features of the patients (Tables S2–S4, Supplemental Digital Content, http://links.lww.com/MD/K861, http://links.lww.com/MD/K862, http://links.lww.com/MD/K863). TPD-L1 expression did not correlate with OS in patients with GAC (*P* = .069; Fig. 2A). However, high IPD-L1 expression was associated with longer OS in patients with GAC (*P* = .028; Fig. 2B). Additionally, patients with high CD8 or CD19 expression showed better OS (*P* = .020 and *P* = .001, respectively; Fig. S1, Supplemental Digital Content, http://links.lww.com/MD/K870).

To better understand the link between PD-L1 and TIICs, the study examined the correlation between the expression of PD-L1 and TIICs markers. Significant correlations were found between TPD-L1 and CD3 (*R* = 0.390, *P* < .001), CD4 (*R* = 0.272, *P* < .001), CD8 (*R* = 0.185, *P* = .002), CD31 (*R* = 0.192, *P* = .002), CD68 (*R* = 0.287, *P* < .001), CD11c (*R* = 0.211, *P* < .001), and α-SMA (r = −0.135, *P* = .028) expression (Table 2). Furthermore, significant correlations were observed between IPD-L1 and CD3 (*R* = 0.341, *P* < .001), CD4 (*R* = 0.282, *P* < .001), CD8 (*R* = 0.269, *P* < .001), CD19 (*R* = 0.352, *P < *.001), CD31 (*R* = 0.302, *P* < .001), CD68 (*R* = 0.266, *P < *.001), and CD11c (*R* = 0.294, *P* < .001) expression (Table 2).

**Table 2:** Correlation between PD-L1 and different immune cells.

	CD3	CD4	CD8	CD19	CD31	CD68	CD11c	CD56	α-SMA
TPD-L1									
r	0.390	0.272	0.185	0.116	0.192	0.287	0.211	−0.093	−0.135
*P*	<.001	<.001	.002	.558	.002	<.001	<.001	.127	.028
IPD-L1									
r	0.341	0.282	0.269	0.352	0.302	0.277	0.294	0.096	0.015
*P*	<.001	<.001	<.001	<.001	<.001	<.001	<.001	.115	.803

**Figure 3.**
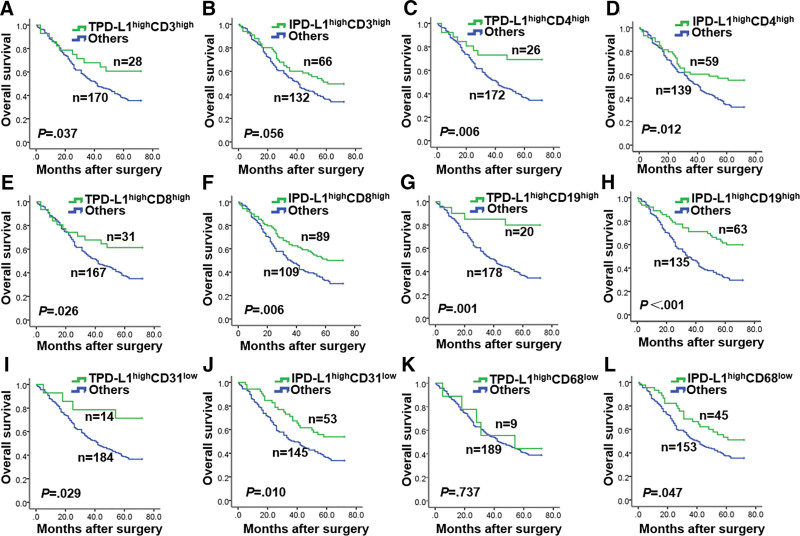
Survival data were analyzed by PD-L1 expression with different immune cells infiltration. (A, C, E, G, I, K) TPD-L1 with CD3, CD4, CD8, CD19, CD31, and CD68 infiltration. (B, D, F, H, J, L) IPD-L1 with CD3, CD4, CD8, CD19, CD31, and CD68 infiltration.

To assess the clinical implications of the findings, the study analyzed the effects of combining different patterns of PD-L1 and TIICs marker expression on patient OS. Survival curve analysis revealed significantly prolonged survival for patients with TPD-L1^high^CD3^high^ expression compared to other groups (*P* = .037; Fig. 3A), whereas there was no significant correlation between IPD-L1^high^CD3^high^ expression and OS (*P* = .056; Fig. 3B). However, patients with PD-L1^high^CD8^high^ expression had a superior OS compared to other groups, irrespective of PD-L1 expression in tumor cells or TIICs (*P* = .026 and *P* = .006, respectively; Fig. 3E and F). Similar results were observed in the TPD-L1^high^CD4^high^ and IPD-L1^high^CD4^high^ groups (*P* = .006 and *P* = .012; Fig. 3C and D) and TPD-L1^high^CD19^high^ and IPD-L1^high^CD19^high^ groups (*P* = .001 and *P* < .001, respectively; Fig. 3G and H). Additionally, survival curve analysis showed significantly longer survival time for patients with PD-L1^high^CD31^low^ expression compared to other groups, regardless of PD-L1 expression in tumor cells or TIICs (*P* = .029 and *P* = .001, Fig. 3I and J). Furthermore, patients with IPD-L1^high^CD68^low^ expression had better prognosis when compared to other groups (*P* = .047; Fig. 3L). However, there was no significant statistical significance between TPD-L1^high^CD68^low^ combination and other groups (*P* = .737; Fig. 3K). No discernible differences were observed when PD-L1 expression was combined with the expression of other TIICs markers (Fig. S2A–F, Supplemental Digital Content, http://links.lww.com/MD/K871). The study also analyzed the relationship between different patterns of PD-L1 expression and the expression of various TIICs markers and clinicopathological features (Tables S5–S10, Supplemental Digital Content, http://links.lww.com/MD/K864, http://links.lww.com/MD/K865, http://links.lww.com/MD/K866, http://links.lww.com/MD/K867, http://links.lww.com/MD/K868, http://links.lww.com/MD/K869).

Univariate and multivariate survival analyses were conducted to examine the association between each index and the survival of patients with GAC. Univariate survival analysis demonstrated that deeper tumor infiltration, increased lymph node involvement, metastasis, higher tumor stage, low IPD-L1 expression, and low levels of CD8 or CD19 expression were high-risk factors for OS (Table 3). Multivariate survival analysis revealed that lymph node involvement, tumor stage, the combination of IPD-L1^high^ and CD8^high^ expression, and the combination of TPD-L1^high^ and CD31^low^ expression were independent factors affecting prognosis (Table 4).

**Table 3:** Univariate analysis of overall survival.

Characteristic	Hazard ratio (95% CI)	*P*
*Tumor depth*		
T1 vs T2 + T3 + T4	0.182 (0.074–0.446)	<.001
*LN involvement*		
N0 vs N1 + N2 + N3	0.296 (0.184–0.475)	<.001
*Metastasis*		
M0 vs M1	0.472 (0.285–0.782)	.004
*Tumor stage*		
0 + I versus II + III + IV	0.110 (0.041–0.299)	<.001
*TPD-L1*		
High versus low	0.626 (0.374–1.045)	.073
*IPD-L1*		
High versus low	0.671 (0.468–0.962)	.030
*CD3*		
High versus low	0.715 (0.491–1.041)	.080
*CD4*		
High versus low	0.715 (0.484–1.057)	.093
*CD8*		
High versus low	0.657 (0.458–0.942)	.022
*CD19*		
High versus low	0.517 (0.348–0.768)	.001
*CD31*		
High versus low	1.198 (0.836–1.717)	.325
*CD68*		
High versus low	1.212 (0.845–1.737)	.296
*IPD-L1 and CD8 expression*		
IPD-L1^high^CD8^high^ versus others*	0.601 (0.414–0.872)	.007
*TPD-L1 and CD31 expression*		
TPD-L1^high^CD31^low^ versus others†	0.348 (0.128–0.945)	.038

* Others = IPD-L1^high^CD8^low^ and IPD-L1^low^CD8^high^ and IPD-L1^low^CD8^low^.

† Others = TPD-L1^high^CD31^high^ and TPD-L1^low^CD31^high^ and TPD-L1^low^CD31^low^.

**Table 4:** Multivariate analysis of overall survival.

Characteristic	Hazard ratio (95% CI)	*P*
*Tumor depth*		
T1 versus T2 + T3 + T4	0.441 (0.170–1.145)	.093
*LN involvement*		
N0 versus N1 + N2 + N3	0.567 (0.337–0.952)	.032
*Metastasis*		
M0 versus M1	0.614 (0.369–1.023)	.061
*Tumor stage*		
0 + I versus II + III + IV	0.268 (0.085–0.846)	.025
*IPD-L1 and CD8 expression*		
IPD-L1^high^CD8^high^ versus others*	0.667 (0.458–0.970)	.034
*TPD-L1 and CD31 expression*		
TPD-L1^high^CD31^low^ versus others†	0.349 (0.128–0.952)	.040

* Others = IPD-L1^high^CD8^low^ and IPD-L1^low^CD8^high^ and IPD-L1^low^CD8^low^.

† Others = TPD-L1^high^CD31^high^ and TPD-L1^low^CD31^high^ and TPD-L1^low^CD31^low^.

## 4. Discussion

In recent years, cancer immunotherapy targeting PD-1/PD-L1 has shown significant progress in the treatment of various tumors and continues to evolve through ongoing clinical research.^[26–28]^ In this study, we found that the significance of PD-L1 expression in tumor and stromal cells remained inconsistent. High PD-L1 expression in tumor cells was associated with larger tumor volume, deeper tumor depth, and poorer tumor differentiation, suggesting an oncogenic role facilitating immune evasion, tumor growth, and invasion. Conversely, high PD-L1 expression in stromal cells was associated with shallower tumor depth and lower mortality rates, indicating a potential activated immune response that enhances immune-mediated tumor killing and inhibits tumor growth. We also observed positive correlations between PD-L1 expression and various immune cells. Multivariate survival analysis revealed that the combination of IPD-L1^high^ and CD8^high^ expression or TPD-L1^high^ and CD31^low^ expression could serve as independent prognostic factors. The immune microenvironment is increasingly recognized for its crucial role in patient prognosis, and combined evaluation of PD-L1 and TIICs expression may aid in better patient selection for immunotherapy.

The tumor-infiltrating immune cells in the tumor tissue primarily include T and B lymphocytes, dendritic cells, macrophages, and mast cells. Previous studies have demonstrated that higher levels of tumor-infiltrating lymphocytes (TILs) are associated with improved prognosis in patients with breast cancer, non-small cell lung cancer, ovarian cancer, and other solid tumors, and the expression of different subsets of TILs correlates with patient survival.^[29–32]^ An animal model of malignant melanoma showed that CD4+ T lymphocyte infiltration could lead to tumor regression. Studies on CD4+ CD25+ regulatory T cells (Tregs TILs) have indicated that increased Treg TILs expression is a high-risk factor for tumor recurrence and is associated with tumor progression.^[33,34]^ Studies in lung cancer patients have revealed that high expression of CD3+ and CD8+ T cells in tumor tissue is associated with better overall survival.^[29,35,36]^ Similarly, a study on gastrointestinal stromal tumors demonstrated that high expression of CD8+ or CD3+ TILs was associated with reduced size of PD-L1/IDO (indoleamine-2,3-dioxygenase) positive tumors.^[37]^ In this study, PD-L1 expression positively correlated with the expression of these immune cells. Patients with high PD-L1 expression and high CD3+, CD4+, or CD8+ T cell expression in tumor tissues showed better prognosis than other subgroups, and the differences were statistically significant. Previous studies have suggested that CD8+ T cells in tumor tissue can upregulate PD-L1 expression through the release of interferon-γ, which may lead to a more favorable response to immunotherapy.^[38]^ In colorectal cancer, the type, density, and location of immune cells infiltrating the tumor site have been proposed to more accurately predict patient survival than the traditional tumor-node-metastasis (TNM) staging.^[35,39]^ Tumors rich in CD3+ and CD8+ T lymphocytes have been classified as “hot tumors,” displaying a more robust immune response to immune checkpoint antibody treatment compared to “cold tumors” lacking lymphocyte infiltration.^[36]^ Additionally, the presence of PD-L1 expression in tumor-related immune cells has been considered a feature of “hot tumors.”^[40]^ In this study, areas with high PD-L1 expression exhibited abundant lymphocyte infiltration. Moreover, the combination of PD-L1 and CD8 expression was identified as an independent prognostic factor. Studies have demonstrated that CD8+ T cells primarily kill tumor cells by releasing cytotoxic factors such as perforin 1 (PRF1), granzyme A (GZMA), granzyme B (GZMB), granzyme H (GZMH).^[41,42]^ Infiltration of CD8+ T lymphocytes into tumor tissue is considered a prerequisite for the successful treatment of metastatic melanoma with anti-PD-1, highlighting the critical role of CD8+ cytotoxic T lymphocytes in antitumor therapy.^[30,43,44]^

In addition to the effects of PD-L1 and T lymphocytes on patient prognosis, we observed a close relationship between PD-L1 and B lymphocyte expression. Our results indicated that PD-L1 expression was consistent with that of CD19, and patients with PD-L1^high^CD19^high^ expression had significantly better prognosis than other combinations, consistent with previous studies.^[45]^ B lymphocytes, a type of tumor-infiltrating immune cell, primarily produce antibodies, present antigens, and secrete immunoglobulins and cytokines. As antigen-presenting cells, B lymphocytes indirectly participate in the cellular immune response, playing an important role in both innate and adaptive immunity.^[46,47]^ Prior studies have shown that gastric cancer patients with high CD19+ B lymphocyte levels in their peripheral blood have longer overall survival.^[48]^ However, the regulatory relationship between PD-L1 and CD19 expression requires further clarification. Elucidating the function and regulatory mechanisms of PD-L1 in T and B lymphocytes could have significant implications for patient treatment.

Notably, Kaplan–Meier survival curve analysis revealed that patients with either low TPD-L1 or IPD-L1 expression along with high CD31 expression had worse prognosis than other groups. Studies have highlighted the importance of angiogenesis in tumor growth and metastasis, and CD31 is involved in the process of cell migration and angiogenesis.^[49,50]^ Mice xenografted with cells overexpressing CD31 displayed significantly shorter survival.^[50]^ Studies have shown that angiogenesis is necessary for tumor growth and metastasis.^[51]^ These findings strongly support the inhibition of tumor angiogenesis by targeting CD31. Our study confirmed that lymphocyte infiltration in tumor tissue is associated with a favorable prognosis, whereas high CD31 expression is a key factor for poor prognosis. Additionally, multivariate survival analysis identified the combination of PD-L1 and CD31 expression as an independent prognostic factor in patients, suggesting a potential new strategy and direction for the treatment of GAC. The combination of an anti-PD-1/PD-L1 monoclonal antibody and an anti-CD31 antibody may yield a more effective therapeutic outcome.

This study does have some limitations. Firstly, since it was a retrospective study conducted in gastric cancer without immune therapy such as PD1 monoclonal antibodies, we didn’t evaluate the impact of differences in patients’ immune microenvironments on the effectiveness of immunotherapy. Secondly, the limited tumor locations selected may not have accurately represented the overall tumor due to intra-tumoral heterogeneity. Nonetheless, these findings hold instructive value and can provide guidance for future prospective clinical trials. Thirdly, the PD-L1 antibody used for the study clone-SP142 had more positive staining on immune cells than on tumor cells. The combined application of multiple PD-L1 antibodies may yield more precise results when examining the relationship between PD-L1 and immune microenvironments.

In conclusion, the tumor immune microenvironment is a complex yet critical factor in cancer biology. Various immune cells and secreted cytokines play pivotal roles in tumor initiation, progression, metastasis, and also influence the therapeutic outcomes of anti-PD-1/PD-L1 treatments, offering hope to patients with diverse tumor types. The findings of this study reveal that the expression of both TPD-L1 and IPD-L1 was associated with significant immune cell infiltration. Combining PD-L1 expression with tumor-infiltrating immune cells can improve patient selection for immunotherapy, and adopting a dual-target strategy (i.e., the combination of anti-PD-1/PD-L1 and anti-CD31 antibodies) can activate effector T cells while inhibiting tumor angiogenesis, potentially enhancing the effectiveness of immunotherapy for GAC.

## Acknowledgments

The authors thank Min Zhang for technical assistance in preparing tissue chips and Dr Liu Mi guidance and help in biostatistics field.

## Author contributions

**Conceptualization:** Lei Cao, Lingchuan Guo.

**Data curation:** Lei Cao, Qiuying Quan, Lili Huang, Zhiju Liu, Tianwei Guo.

**Formal analysis:** Lei Cao, Qiuying Quan, Lili Huang, Zhiju Liu, Tianwei Guo.

**Funding acquisition:** Lei Cao, Lingchuan Guo.

**Investigation:** Qiuying Quan, Lili Huang.

**Methodology:** Lei Cao.

**Project administration:** Lei Cao, Lingchuan Guo.

**Resources:** Qiuying Quan, Lili Huang, Zhiju Liu.

**Software:** Lei Cao.

**Supervision:** Lei Cao, Lingchuan Guo.

**Validation:** Lei Cao, Lingchuan Guo.

**Visualization:** Lei Cao, Lingchuan Guo.

**Writing – original draft:** Qiuying Quan, Lili Huang.

**Writing – review & editing:** Lei Cao, Yu Shen, Sisi Ding, Cuiping Liu, Lingchuan Guo.

## Supplementary Material
























