# Supplementary material for: Expression and clinical significance of PD-L1 and infiltrated immune cells in the gastric adenocarcinoma microenvironment
Source: Medicine (Baltimore). 2023 Dec 1;102(48):e36323. doi: 10.1097/MD.0000000000036323 (PMC10695517; doi:10.1097/MD.0000000000036323)
Supplement: Supplementary file 1 [file medi-102-e36323-s001.docx]

**Table S1:** Clinicopathologic features of the GAC patients

| Clinicopathologic features | | Number (%) |
| --- | --- | --- |
| Age |  |  |
| Range, median (years) | | 33-88, 65 |
| Sex |  |  |
| Female |  | 58 (27.6%) |
| Male |  | 210 (72.4%) |
| Tumor volume (cm^3^) | |  |
| <5 |  | 186 (69.4%) |
| ≥5 |  | 82 (30.60%) |
| Tumor differentiation | |  |
| well |  | 6 (2.24%) |
| moderate | | 121 (45.15%) |
| poor |  | 141 (52.61%) |
| Tumor depth | |  |
| T1 |  | 36 (13.43%) |
| T2+T3+T4 | | 232 (86.57%) |
| Nodal status | |  |
| N0 |  | 85 (31.72%) |
| N1+N2+N3 | | 183 (68.28%) |
| Distant metastasis | |  |
| M0 |  | 238 (88.81%) |
| M1 |  | 30 (11.19%) |
| Tumor stage | |  |
| 0+I |  | 43 (16.04%) |
| II+III+IV | | 225 (83.96%) |
| Status |  |  |
| Dead |  | 120 (60.61%) |
| Alive |  | 78 (39.39%) |
| Overall survival | |  |
| (range, median) | | 1-72, 43.5 |
