# Supplementary material for: Expression and clinical significance of PD-L1 and infiltrated immune cells in the gastric adenocarcinoma microenvironment
Source: Medicine (Baltimore). 2023 Dec 1;102(48):e36323. doi: 10.1097/MD.0000000000036323 (PMC10695517; doi:10.1097/MD.0000000000036323)
Supplement: Supplementary file 2 [file medi-102-e36323-s002.docx]

**Table S2:** The relationship between CD3, CD4, CD8 expression and clinical pathological features

| Clinical or pathologic  Factors | Total  No. | CD3 | | *P* | CD4 | | *P* | CD8 | | *P* |
| --- | --- | --- | --- | --- | --- | --- | --- | --- | --- | --- |
|  |  | low | high |  | low | high |  | low | high |  |
| All cases | 268 | 160 | 108 |  | 165 | 103 |  | 106 | 162 |  |
| Age |  |  |  | .817 |  |  | 0.994 | 58 | 106 | .079 |
| ＜70 | 164 | 97 | 67 |  | 101 | 63 |  | 48 | 56 |  |
| ≥70 | 104 | 63 | 41 |  | 64 | 40 |  |  |  |  |
| Sex |  |  |  | .679 |  |  | 0.695 |  |  | .035 |
| Female | 58 | 36 | 22 |  | 37 | 21 |  | 16 | 42 |  |
| Male | 210 | 124 | 86 |  | 128 | 82 |  | 90 | 120 |  |
| Tumor volume（cm3） |  |  |  | .797 |  |  | 0.223 |  |  | .335 |
| ＜5 | 186 | 112 | 74 |  | 119 | 67 |  | 70 | 116 |  |
| ≥5 | 82 | 48 | 34 |  | 46 | 36 |  | 36 | 46 |  |
| Tumor differentiation |  |  |  | .293 |  |  | 0.158 |  |  | .583 |
| Well | 6 | 4 | 2 |  | 4 | 2 |  | 2 | 4 |  |
| Moderate | 121 | 76 | 45 |  | 80 | 41 |  | 51 | 70 |  |
| Poor | 141 | 80 | 61 |  | 81 | 60 |  | 53 | 88 |  |
| Tumor depth |  |  |  | .854 |  |  | 0.032 |  |  | .022 |
| T1 | 36 | 22 | 14 |  | 28 | 8 |  | 8 | 28 |  |
| T2+T3+T4 | 232 | 138 | 94 |  | 137 | 95 |  | 98 | 134 |  |
| LN involvement |  |  |  | .548 |  |  | 0.127 |  |  | .665 |
| N0 | 85 | 53 | 32 |  | 58 | 27 |  | 32 | 53 |  |
| N1+N2+N3 | 183 | 107 | 76 |  | 107 | 76 |  | 74 | 109 |  |
| Metastasis |  |  |  | .792 |  |  | 0.327 |  |  | .958 |
| M0 | 238 | 142 | 96 |  | 149 | 89 |  | 94 | 144 |  |
| M1 | 30 | 18 | 12 |  | 16 | 14 |  | 12 | 18 |  |
| Tumor stage |  |  |  | .654 |  |  | 0.389 |  |  | .174 |
| 0+I | 43 | 27 | 16 |  | 29 | 14 |  | 13 | 30 |  |
| II+III+IV | 225 | 133 | 92 |  | 136 | 89 |  | 93 | 132 | .029 |
| Death |  |  |  | .082 |  |  | 0.033 |  |  |  |
| No | 78 | 41 | 37 |  | 43 | 35 |  | 23 | 55 |  |
| Yes | 120 | 78 | 42 |  | 84 | 36 |  | 54 | 66 |  |
