# Supplementary material for: Expression and clinical significance of PD-L1 and infiltrated immune cells in the gastric adenocarcinoma microenvironment
Source: Medicine (Baltimore). 2023 Dec 1;102(48):e36323. doi: 10.1097/MD.0000000000036323 (PMC10695517; doi:10.1097/MD.0000000000036323)
Supplement: Supplementary file 3 [file medi-102-e36323-s003.docx]

**Table S3:** The relationship between CD19, CD31, CD68 expression and clinical pathological features

| Clinical or pathologic  Factors | Total  No. | CD19 | | *P* | CD31 | | *P* | CD68 | | *P* |
| --- | --- | --- | --- | --- | --- | --- | --- | --- | --- | --- |
|  |  | low | high |  | low | high |  | low | high |  |
| All cases | 268 | 155 | 113 |  | 150 | 118 |  | 130 | 138 |  |
| Age |  |  |  | .046 |  |  | 0.145 |  |  | .541 |
| ＜70 | 164 | 87 | 77 |  | 86 | 78 |  | 82 | 82 |  |
| ≥70 | 104 | 68 | 36 |  | 64 | 40 |  | 48 | 56 |  |
| Sex |  |  |  | .301 |  |  | 0.176 |  |  | .798 |
| Female | 58 | 37 | 21 |  | 37 | 21 |  | 29 | 29 |  |
| Male | 210 | 118 | 92 |  | 113 | 97 |  | 101 | 109 |  |
| Tumor volume（cm3） |  |  |  | .021 |  |  | 0.978 |  |  | .318 |
| ＜5 | 186 | 99 | 87 |  | 104 | 82 |  | 94 | 92 |  |
| ≥5 | 82 | 56 | 26 |  | 46 | 36 |  | 36 | 46 |  |
| Tumor differentiation |  |  |  | .167 |  |  | 0.921 |  |  | .216 |
| Well | 6 | 3 | 3 |  | 4 | 2 |  | 2 | 4 |  |
| Moderate | 121 | 77 | 44 |  | 66 | 55 |  | 55 | 66 |  |
| Poor | 141 | 75 | 66 |  | 80 | 61 |  | 73 | 68 |  |
| Tumor depth |  |  |  | ＜.001 |  |  | 0.081 |  |  | .001 |
| T1 | 36 | 8 | 28 |  | 25 | 11 |  | 27 | 9 |  |
| T2+T3+T4 | 232 | 147 | 85 |  | 125 | 107 |  | 103 | 29 |  |
| LN involvement |  |  |  | .030 |  |  | 0.026 |  |  | .010 |
| N0 | 85 | 41 | 44 |  | 56 | 29 |  | 51 | 34 |  |
| N1+N2+N3 | 183 | 114 | 69 |  | 94 | 89 |  | 79 | 104 |  |
| Metastasis |  |  |  | .300 |  |  | 0.759 |  |  | .170 |
| M0 | 238 | 135 | 103 |  | 134 | 104 |  | 119 | 119 |  |
| M1 | 30 | 20 | 10 |  | 16 | 14 |  | 11 | 19 |  |
| Tumor stage |  |  |  | .003 |  |  | 0.099 |  |  | ＜.001 |
| 0+I | 43 | 16 | 27 |  | 29 | 14 |  | 34 | 9 |  |
| II+III+IV | 225 | 139 | 86 |  | 121 | 104 |  | 96 | 129 |  |
| Death |  |  |  | .001 |  |  | 0.366 |  |  | .390 |
| No | 78 | 37 | 41 |  | 48 | 30 |  | 40 | 38 |  |
| Yes | 120 | 85 | 35 |  | 66 | 54 |  | 54 | 66 |  |
