# Supplementary material for: Expression and clinical significance of PD-L1 and infiltrated immune cells in the gastric adenocarcinoma microenvironment
Source: Medicine (Baltimore). 2023 Dec 1;102(48):e36323. doi: 10.1097/MD.0000000000036323 (PMC10695517; doi:10.1097/MD.0000000000036323)
Supplement: Supplementary file 4 [file medi-102-e36323-s004.docx]

**Table S4:** The relationship between CD11c, CD56 and α-SMA expression and clinical pathological features

| Clinical or pathologic  Factors | Total  No. | CD11c | | *P* | CD56 | | *P* | α-SMA | | *P* |
| --- | --- | --- | --- | --- | --- | --- | --- | --- | --- | --- |
|  |  | low | high |  | low | high |  | low | high |  |
| All cases | 268 | 135 | 133 |  | 236 | 32 |  |  |  |  |
| Age |  |  |  | .923 |  |  | .823 |  |  | .465 |
| ＜70 | 164 | 83 | 81 |  | 145 | 19 |  | 17 | 147 |  |
| ≥70 | 104 | 52 | 52 |  | 91 | 13 |  | 8 | 96 |  |
| Sex |  |  |  | .044 |  |  | .673 |  |  | .835 |
| Female | 58 | 36 | 22 |  | 52 | 6 |  | 5 | 53 |  |
| Male | 210 | 99 | 111 |  | 184 | 26 |  | 20 | 190 |  |
| Tumor volume（cm3） |  |  |  | .730 |  |  | .122 |  |  | .001 |
| ＜5 | 186 | 95 | 91 |  | 160 | 26 |  | 10 | 176 |  |
| ≥5 | 82 | 40 | 42 |  | 76 | 6 |  | 15 | 67 |  |
| Tumor differentiation |  |  |  | .502 |  |  | .179 |  |  | .354 |
| Well | 6 | 2 | 4 |  | 6 | 0 |  | 0 | 6 |  |
| Moderate | 121 | 60 | 61 |  | 109 | 12 |  | 10 | 111 |  |
| Poor | 141 | 73 | 68 |  | 121 | 20 |  | 15 | 126 |  |
| Tumor depth |  |  |  | .014 |  |  | .009 |  |  | .039 |
| T1 | 36 | 25 | 11 |  | 27 | 9 |  | 0 | 36 |  |
| T2+T3+T4 | 232 | 110 | 122 |  | 209 | 23 |  | 25 | 207 |  |
| LN involvement |  |  |  | .175 |  |  | .005 |  |  | .676 |
| N0 | 85 | 48 | 37 |  | 68 | 17 |  | 7 | 78 |  |
| N1+N2+N3 | 183 | 87 | 96 |  | 168 | 15 |  | 18 | 165 |  |
| Metastasis |  |  |  | .229 |  |  |  |  |  | .894 |
| M0 | 238 | 123 | 115 |  | 209 | 29 | .729 | 22 | 216 |  |
| M1 | 30 | 12 | 18 |  | 27 | 3 |  | 3 | 27 |  |
| Tumor stage |  |  |  | .035 |  |  | ＜.001 |  |  | .085 |
| 0+I | 43 | 28 | 15 |  | 31 | 12 |  | 1 | 42 |  |
| II+III+IV | 225 | 107 | 118 |  | 205 | 20 |  | 24 | 207 |  |
| Death |  |  |  | .532 |  |  | .878 |  |  | .898 |
| No | 78 | 40 | 38 |  | 69 | 9 |  | 8 | 70 |  |
| Yes | 120 | 67 | 53 |  | 107 | 13 |  | 13 | 107 |  |
