# Supplementary figures and images for: Expression and clinical significance of PD-L1 and infiltrated immune cells in the gastric adenocarcinoma microenvironment
Source: Medicine (Baltimore). 2023 Dec 1;102(48):e36323. doi: 10.1097/MD.0000000000036323 (PMC10695517; doi:10.1097/MD.0000000000036323)

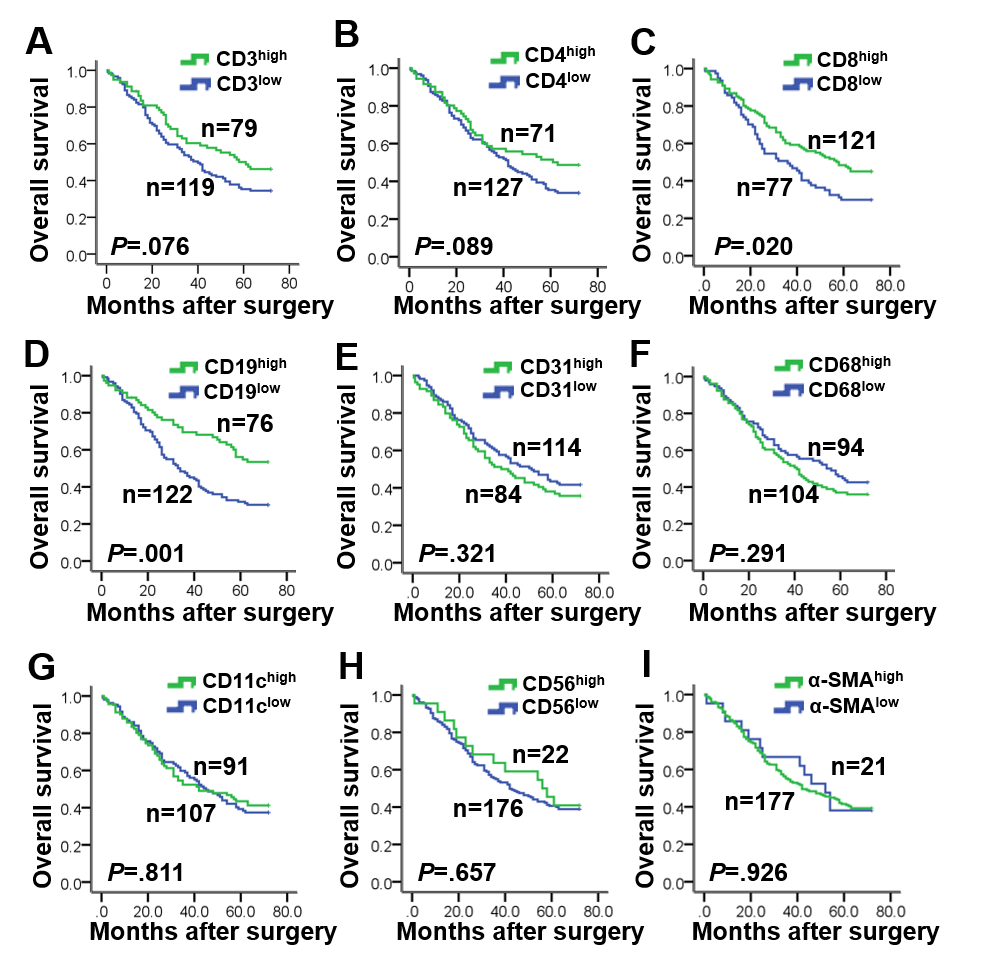

Supplement: Supplementary file 5 [file medi-102-e36323-s005.tif]

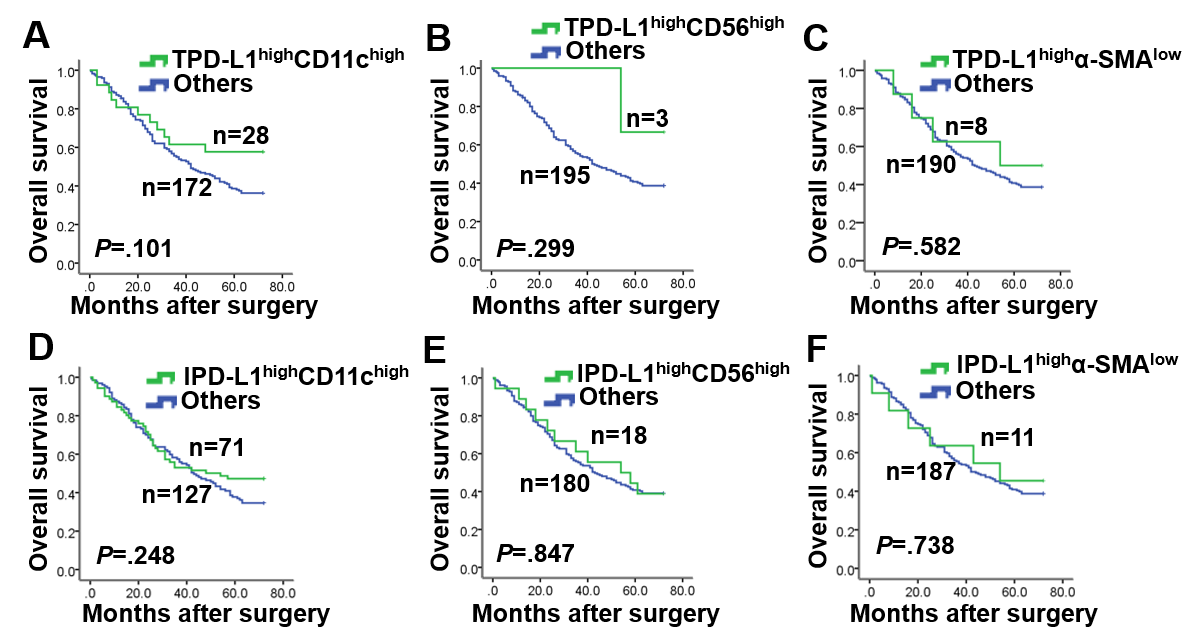

Supplement: Supplementary file 6 [file medi-102-e36323-s006.tif]
