# Supplementary material for: Expression and clinical significance of PD-L1 and infiltrated immune cells in the gastric adenocarcinoma microenvironment
Source: Medicine (Baltimore). 2023 Dec 1;102(48):e36323. doi: 10.1097/MD.0000000000036323 (PMC10695517; doi:10.1097/MD.0000000000036323)
Supplement: Supplementary file 7 [file medi-102-e36323-s007.docx]

**Table S5:** The relationship between combination of PD-L1 and CD3 expression and clinicopathological features

| Clinicopathologic Factors | Total No | TPDL1 and CD3 combination | | *P* | IPDL1 and CD3 combination | | *P* |
| --- | --- | --- | --- | --- | --- | --- | --- |
|  |  | Others^†^ | TPDL1^high^CD3^high^ |  | Others^‡^ | IPDL1^high^CD3^high^ |  |
| All cases | 268 | 231 | 37 |  | 179 | 89 |  |
| Age |  |  |  | .393 |  |  | .501 |
| ﹤70 | 164 | 139 | 25 |  | 107 | 57 |  |
| ≥70 | 104 | 92 | 12 |  | 72 | 32 |  |
| Sex |  |  |  | .997 |  |  | .098 |
| Female | 58 | 50 | 8 |  | 44 | 14 |  |
| Male | 210 | 181 | 29 |  | 135 | 75 |  |
| Tumor volume (cm^3^) |  |  |  | .073 |  |  | .730 |
| ﹤5 | 186 | 165 | 21 |  | 123 | 63 |  |
| ≥5 | 82 | 66 | 16 |  | 56 | 26 |  |
| Tumor differentiation |  |  |  | .002 |  |  | .606 |
| Well | 6 | 6 | 0 |  | 4 | 2 |  |
| Moderate | 121 | 112 | 9 |  | 83 | 38 |  |
| Poor | 141 | 113 | 28 |  | 92 | 49 |  |
| Tumor depth |  |  |  | .010 |  |  | .986 |
| T1 | 36 | 36 | 0 |  | 24 | 12 |  |
| T2+T3+T4 | 232 | 195 | 37 |  | 155 | 77 |  |
| LN involvement |  |  |  | .511 |  |  | .950 |
| N0 | 85 | 75 | 10 |  | 57 | 28 |  |
| N1+N2+N3 | 183 | 156 | 27 |  | 122 | 61 |  |
| Metastasis |  |  |  | .631 |  |  | .988 |
| M0 | 238 | 206 | 32 |  | 159 | 79 |  |
| M1 | 30 | 25 | 5 |  | 20 | 10 |  |
| Tumor stage |  |  |  | .058 |  |  | .922 |
| 0+I | 43 | 41 | 2 |  | 29 | 14 |  |
| II+III+IV | 225 | 190 | 35 |  | 150 | 75 |  |
| Death |  |  |  | .013 |  |  | .031 |
| No | 78 | 61 | 17 |  | 45 | 33 |  |
| Yes | 120 | 109 | 11 |  | 87 | 33 |  |

Others^†^ = TPDL1^high^CD3^low^ and TPDL1^low^CD3^high^ and TPDL1^low^CD3^low^.

Others^‡^ = IPDL1^high^CD3^low^ and IPDL1^low^CD3^high^ and IPDL1^low^CD3^low^.
