# Supplementary material for: Expression and clinical significance of PD-L1 and infiltrated immune cells in the gastric adenocarcinoma microenvironment
Source: Medicine (Baltimore). 2023 Dec 1;102(48):e36323. doi: 10.1097/MD.0000000000036323 (PMC10695517; doi:10.1097/MD.0000000000036323)
Supplement: Supplementary file 8 [file medi-102-e36323-s008.docx]

**Table S6:** The relationship between combination of PD-L1 and CD4 expression and clinicopathological features

| Clinicopathologic Factors | Total No | TPDL1 and CD4 combination | | *P* | IPDL1 and CD4 combination | | *P* |
| --- | --- | --- | --- | --- | --- | --- | --- |
|  |  | Others^※^ | TPDL1^high^CD4^high^ |  | Others^#^ | IPDL1^high^CD4^high^ |  |
| All cases | 268 | 234 | 34 |  | 186 | 82 |  |
| Age |  |  |  | .942 |  |  | .961 |
| ﹤70 | 164 | 143 | 21 |  | 114 | 50 |  |
| ≥70 | 104 | 91 | 13 |  | 72 | 32 |  |
| Sex |  |  |  | .776 |  |  | .229 |
| Female | 58 | 50 | 8 |  | 44 | 14 |  |
| Male | 210 | 184 | 26 |  | 142 | 68 |  |
| Tumor volume (cm^3^) |  |  |  | .008 |  |  | .404 |
| ﹤5 | 186 | 169 | 17 |  | 132 | 54 |  |
| ≥5 | 82 | 65 | 17 |  | 54 | 28 |  |
| Tumor differentiation |  |  |  | .020 |  |  | .681 |
| Well | 6 | 6 | 0 |  | 4 | 2 |  |
| Moderate | 121 | 111 | 10 |  | 86 | 35 |  |
| Poor | 141 | 117 | 24 |  | 96 | 45 |  |
| Tumor depth |  |  |  | .014 |  |  | .119 |
| T1 | 36 | 36 | 0 |  | 29 | 7 |  |
| T2+T3+T4 | 232 | 198 | 34 |  | 157 | 75 |  |
| LN involvement |  |  |  | .758 |  |  | .569 |
| N0 | 85 | 75 | 10 |  | 61 | 24 |  |
| N1+N2+N3 | 183 | 159 | 24 |  | 125 | 58 |  |
| Metastasis |  |  |  | .489 |  |  | .731 |
| M0 | 238 | 209 | 29 |  | 166 | 72 |  |
| M1 | 30 | 25 | 5 |  | 20 | 10 |  |
| Tumor stage |  |  |  | .085 |  |  | .677 |
| 0+I | 43 | 41 | 2 |  | 31 | 12 |  |
| II+III+IV | 225 | 193 | 32 |  | 155 | 70 |  |
| Death |  |  |  | .001 |  |  | .002 |
| No | 78 | 60 | 18 |  | 45 | 33 |  |
| Yes | 120 | 112 | 8 |  | 94 | 26 |  |

Others^※^ = TPDL1^high^CD4^low^ and TPDL1^low^CD4^high^ and TPDL1^low^CD4^low^.

Others^#^ = IPDL1^high^CD4^low^ and IPDL1^low^CD4^high^ and IPDL1^low^CD4^low^.
