# Supplementary material for: Expression and clinical significance of PD-L1 and infiltrated immune cells in the gastric adenocarcinoma microenvironment
Source: Medicine (Baltimore). 2023 Dec 1;102(48):e36323. doi: 10.1097/MD.0000000000036323 (PMC10695517; doi:10.1097/MD.0000000000036323)
Supplement: Supplementary file 9 [file medi-102-e36323-s009.docx]

**Table S7:** The relationship between combination of PD-L1 and CD8 expression and clinicopathological features

| Clinicopathologic Factors | Total  No | TPDL1 and CD8 combination | | *P* | IPDL1 and CD8 combination | | *P* |
| --- | --- | --- | --- | --- | --- | --- | --- |
|  |  | Others^*^ | TPDL1^high^CD8^high^ |  | Others^※^ | IPDL1^high^CD8^high^ |  |
| All cases | 268 | 227 | 41 |  | 150 | 118 |  |
| Age |  |  |  | .313 |  |  | .145 |
| ﹤70 | 164 | 136 | 28 |  | 86 | 78 |  |
| ≥70 | 104 | 91 | 13 |  | 64 | 40 |  |
| Sex |  |  |  | .644 |  |  | .450 |
| Female | 58 | 48 | 10 |  | 35 | 23 |  |
| Male | 210 | 179 | 31 |  | 115 | 95 |  |
| Tumor volume (cm^3^) |  |  |  | .045 |  |  | .409 |
| ﹤5 | 186 | 163 | 23 |  | 101 | 85 |  |
| ≥5 | 82 | 64 | 18 |  | 49 | 33 |  |
| Tumor differentiation |  |  |  | .003 |  |  | .582 |
| Well | 6 | 6 | 0 |  | 2 | 4 |  |
| Moderate | 121 | 110 | 11 |  | 68 | 53 |  |
| Poor | 141 | 111 | 30 |  | 80 | 61 |  |
| Tumor depth |  |  |  | .081 |  |  | .001 |
| T1 | 36 | 34 | 2 |  | 11 | 25 |  |
| T2+T3+T4 | 232 | 193 | 39 |  | 139 | 93 |  |
| LN involvement |  |  |  | .275 |  |  | .142 |
| N0 | 85 | 75 | 10 |  | 42 | 43 |  |
| N1+N2+N3 | 183 | 152 | 31 |  | 108 | 75 |  |
| Metastasis |  |  |  | .826 |  |  | .639 |
| M0 | 238 | 202 | 36 |  | 132 | 106 |  |
| M1 | 30 | 25 | 5 |  | 18 | 12 |  |
| Tumor stage |  |  |  | .099 |  |  | .007 |
| 0+I | 43 | 40 | 3 |  | 16 | 27 |  |
| II+III+IV | 225 | 187 | 38 |  | 134 | 91 |  |
| Death |  |  |  | .006 |  |  | .004 |
| No | 78 | 59 | 19 |  | 33 | 45 |  |
| Yes | 120 | 108 | 12 |  | 76 | 44 |  |

Others* = TPDL1^high^CD8^low^ and TPDL1^low^CD8^high^ and TPDL1^low^CD8^low^.

Others^※^ = IPDL1^high^CD8^low^ and IPDL1^low^CD8^high^ and IPDL1^low^CD8^low^.
