# Supplementary material for: Expression and clinical significance of PD-L1 and infiltrated immune cells in the gastric adenocarcinoma microenvironment
Source: Medicine (Baltimore). 2023 Dec 1;102(48):e36323. doi: 10.1097/MD.0000000000036323 (PMC10695517; doi:10.1097/MD.0000000000036323)
Supplement: Supplementary file 10 [file medi-102-e36323-s010.docx]

**Table S8:** The relationship between combination of PD-L1 and CD19 expression and clinicopathological features

| Clinicopathologic Factors | Total No | TPDL1 and CD19 combination | | *P* | IPDL1 and CD19 combination | | | *P* |
| --- | --- | --- | --- | --- | --- | --- | --- | --- |
|  |  | Others^※^ | TPDL1^high^CD19^high^ |  | | Others^#^ | IPDL1^high^CD19^high^ |  |
| All cases | 268 | 240 | 28 |  | |  |  |  |
| Age |  |  |  | .724 | |  |  | .181 |
| ﹤70 | 164 | 146 | 18 |  | | 102 | 62 |  |
| ≥70 | 104 | 94 | 10 |  | | 73 | 31 |  |
| Sex |  |  |  | .609 | |  |  | .111 |
| Female | 58 | 53 | 5 |  | | 43 | 15 |  |
| Male | 210 | 187 | 23 |  | | 132 | 78 |  |
| Tumor volume (cm^3^) |  |  |  | .807 | |  |  | .018 |
| ﹤5 | 186 | 166 | 20 |  | | 113 | 73 |  |
| ≥5 | 82 | 74 | 8 |  | | 62 | 20 |  |
| Tumor differentiation |  |  |  | .030 | |  |  | .612 |
| Well | 6 | 6 | 0 |  | | 3 | 3 |  |
| Moderate | 121 | 113 | 8 |  | | 83 | 38 |  |
| Poor | 141 | 121 | 20 |  | | 89 | 52 |  |
| Tumor depth |  |  |  | .107 | |  |  | ＜.001 |
| T1 | 36 | 35 | 1 |  | | 13 | 23 |  |
| T2+T3+T4 | 232 | 205 | 27 |  | | 162 | 70 |  |
| LN involvement |  |  |  | .959 | |  |  | .019 |
| N0 | 85 | 76 | 9 |  | | 47 | 38 |  |
| N1+N2+N3 | 183 | 164 | 19 |  | | 128 | 55 |  |
| Metastasis |  |  |  | .585 | |  |  | .568 |
| M0 | 238 | 214 | 24 |  | | 154 | 84 |  |
| M1 | 30 | 26 | 4 |  | | 21 | 9 |  |
| Tumor stage |  |  |  | .419 | |  |  | .005 |
| 0+I | 43 | 40 | 3 |  | | 20 | 23 |  |
| II+III+IV | 225 | 200 | 25 |  | | 155 | 70 |  |
| Death |  |  |  | ＜.001 | |  |  | ＜.001 |
| No | 78 | 62 | 16 |  | | 40 | 38 |  |
| Yes | 120 | 116 | 4 |  | | 95 | 25 |  |

Others^※^ = TPDL1^high^CD19^low^ and TPDL1^low^CD19^high^ and TPDL1^low^CD19^low^.

Others^#^= IPDL1^high^CD19^low^ and IPDL1^low^CD19^high^ and IPDL1^low^CD19^low^.
