# Supplementary material for: Expression and clinical significance of PD-L1 and infiltrated immune cells in the gastric adenocarcinoma microenvironment
Source: Medicine (Baltimore). 2023 Dec 1;102(48):e36323. doi: 10.1097/MD.0000000000036323 (PMC10695517; doi:10.1097/MD.0000000000036323)
Supplement: Supplementary file 11 [file medi-102-e36323-s011.docx]

**Table S9:** The relationship between combination of PD-L1 and CD31 expression and clinicopathological features

| Clinicopathologic Factors | Total No | TPDL1 and CD31 combination | | *P* | IPDL1 and CD31 combination | | *P* |
| --- | --- | --- | --- | --- | --- | --- | --- |
|  |  | Others^‡^ | TPDL1^high^CD31^low^ |  | Others^§^ | IPDL1^high^CD31^low^ |  |
| All cases | 268 | 249 | 19 |  | 194 | 74 |  |
| Age |  |  |  | .504 |  |  | .231 |
| ﹤70 | 164 | 151 | 13 |  | 123 | 41 |  |
| ≥70 | 104 | 98 | 6 |  | 71 | 33 |  |
| Sex |  |  |  | .522 |  |  | .996 |
| Female | 58 | 55 | 3 |  | 42 | 16 |  |
| Male | 210 | 194 | 16 |  | 152 | 58 |  |
| Tumor volume (cm^3^) |  |  |  | .542 |  |  | .435 |
| ﹤5 | 186 | 174 | 12 |  | 132 | 54 |  |
| ≥5 | 82 | 75 | 7 |  | 62 | 20 |  |
| Tumor differentiation |  |  |  | .052 |  |  | .749 |
| Well | 6 | 6 | 0 |  | 4 | 2 |  |
| Moderate | 121 | 116 | 5 |  | 87 | 34 |  |
| Poor | 141 | 127 | 14 |  | 103 | 38 |  |
| Tumor depth |  |  |  | .701 |  |  | ＜.001 |
| T1 | 36 | 34 | 2 |  | 14 | 22 |  |
| T2+T3+T4 | 232 | 215 | 17 |  | 180 | 52 |  |
| LN involvement |  |  |  | .620 |  |  | ＜.001 |
| N0 | 85 | 78 | 7 |  | 49 | 36 |  |
| N1+N2+N3 | 183 | 171 | 12 |  | 145 | 38 |  |
| Metastasis |  |  |  | .924 |  |  | .324 |
| M0 | 238 | 221 | 17 |  | 170 | 68 |  |
| M1 | 30 | 28 | 2 |  | 24 | 6 |  |
| Tumor stage |  |  |  | .498 |  |  | .008 |
| 0+I | 43 | 41 | 2 |  | 24 | 19 |  |
| II+III+IV | 225 | 208 | 17 |  | 170 | 55 |  |
| Death |  |  |  | .011 |  |  | .007 |
| No | 78 | 68 | 10 |  | 49 | 29 |  |
| Yes | 120 | 116 | 4 |  | 96 | 24 |  |

Others^‡^ = TPDL1^high^CD31^high^ and TPDL1^low^CD31^high^ and TPDL1^low^CD31^low^.

Others^§^ = IPDL1^high^CD31^high^ and IPDL1^low^CD31^high^ and IPDL1^low^CD31^low^.
