# Supplementary material for: Expression and clinical significance of PD-L1 and infiltrated immune cells in the gastric adenocarcinoma microenvironment
Source: Medicine (Baltimore). 2023 Dec 1;102(48):e36323. doi: 10.1097/MD.0000000000036323 (PMC10695517; doi:10.1097/MD.0000000000036323)
Supplement: Supplementary file 12 [file medi-102-e36323-s012.docx]

**Table S10:** The relationship between combination of PD-L1 and CD68 expression and clinicopathological features

| Clinicopathologic Factors | Total  No | TPDL1 and CD68 combination | | *P* | IPDL1 and CD68 combination | | *P* |
| --- | --- | --- | --- | --- | --- | --- | --- |
|  |  | Others^※^ | TPDL1^high^CD68^low^ |  | Others^‡^ | IPDL1^high^CD68^low^ |  |
| All cases | 268 | 258 | 10 |  |  |  |  |
| Age |  |  |  | .562 |  |  | .670 |
| ﹤70 | 164 | 157 | 7 |  | 124 | 40 |  |
| ≥70 | 104 | 101 | 3 |  | 81 | 23 |  |
| Sex |  |  |  | .091 |  |  | .205 |
| Female | 58 | 58 | 0 |  | 48 | 10 |  |
| Male | 210 | 200 | 10 |  | 157 | 53 |  |
| Tumor volume (cm^3^) |  |  |  | .513 |  |  | .100 |
| ﹤5 | 186 | 180 | 6 |  | 137 | 49 |  |
| ≥5 | 82 | 78 | 4 |  | 68 | 14 |  |
| Tumor differentiation |  |  |  | .569 |  |  | .323 |
| Well | 6 | 6 | 0 |  | 4 | 2 |  |
| Moderate | 121 | 117 | 4 |  | 90 | 31 |  |
| Poor | 141 | 135 | 6 |  | 111 | 30 |  |
| Tumor depth |  |  |  | .537 |  |  | ＜.001 |
| T1 | 36 | 34 | 2 |  | 2 | 24 |  |
| T2+T3+T4 | 232 | 224 | 8 |  | 193 | 39 |  |
| LN involvement |  |  |  | .906 |  |  | ＜.001 |
| N0 | 85 | 82 | 3 |  | 51 | 34 |  |
| N1+N2+N3 | 183 | 176 | 7 |  | 154 | 29 |  |
| Metastasis |  |  |  | .903 |  |  | .164 |
| M0 | 238 | 229 | 9 |  | 179 | 59 |  |
| M1 | 30 | 29 | 1 |  | 26 | 4 |  |
| Tumor stage |  |  |  | .730 |  |  | ＜.001 |
| 0+I | 43 | 41 | 2 |  | 18 | 25 |  |
| II+III+IV | 225 | 217 | 8 |  | 187 | 38 |  |
| Death |  |  |  | .752 |  |  | .068 |
| No | 78 | 74 | 4 |  | 55 | 23 |  |
| Yes | 120 | 115 | 5 |  | 98 | 22 |  |

Others^※^= TPDL1^high^CD68^high^ and TPDL1^low^CD68^high^ and TPDL1^low^CD68^low^.

Others^‡^ = IPDL1^high^CD68^high^ and IPDL1^low^CD68^high^ and IPDL1^low^CD68^low^.
